# Case Report: Metagenomic next-generation sequencing assists in dynamic pathogen monitoring: powerful tool for progressing severe pneumonia

**DOI:** 10.3389/fcimb.2023.1230813

**Published:** 2023-09-04

**Authors:** Yaoguang Li, Jun Lei, Zhigang Ren, Xiaoxu Ma

**Affiliations:** ^1^ Gene Hospital of Henan Province, Precision Medicine Center, The First Affiliated Hospital of Zhengzhou University, Zhengzhou, China; ^2^ Department of Infectious Disease, The First Affiliated Hospital of Zhengzhou University, Zhengzhou, China; ^3^ Department of Respiration, The First Affiliated Hospital of Zhengzhou University, Zhengzhou, China

**Keywords:** metagenomic next-generation sequencing, pathogen detection, influenza, community-acquired pneumonia, extracorporeal membrane oxygenation, case report

## Abstract

**Background:**

Severe community-acquired pneumonia (sCAP) is life-threatening and characterized by intensive care unit (ICU) admission and high mortality. And they are vulnerable to hospital-acquired infection. In such a severe condition, metagenomic next-generation sequencing (mNGS) outperforms for short turnaround time and broad detection spectrum.

**Case presentation:**

A 15-year-old male with severe influenza and methicillin-resistant *Staphylococcus aureus* (MRSA) pneumonia progressed rapidly, initially misdiagnosed as influenza co-infected with Aspergillus for misleading bronchoscopy manifestations. The turnaround time of mNGS is 13 h, which has the potential to expedite the clinical medication process. With the powerful support of mNGS and extracorporeal membrane oxygenation (ECMO), anti-infective therapy was adjusted accordingly, and vital signs gradually stabilized. After tortuous treatment and unremitting efforts, the patient recovered well.

**Conclusions:**

Rapid mNGS applications, timely medication adjustments, strong ECMO support and active family compliance contribute to this miracle of life. False-negative or false-positive results are alarming, anti-infective medications should be adjusted after a comprehensive review of physical status and other indicators.

## Introduction

Severe community-acquired pneumonia (sCAP) is the most dangerous form of community-acquired pneumonia (CAP) (Niederman and Torres, 2022). CAP is mainly caused by *Streptococcus pneumoniae* (Said et al., 2013) and some respiratory viruses, including influenza virus and rhinovirus (Jain et al., 2015), 21% of the CAP patients required intensive care unit (ICU) admission (Cavallazzi et al., 2020). Unfortunately, these patients are also susceptible to hospital-acquired infection during ICU stay (Markwart et al., 2020). The tortuous treatment course and complicated medication adjustment always result in prolonged hospitalization and unaffordable expenditure (Niederman and Torres, 2022). Even with active treatment, the in-hospital mortality remains high (Cavallazzi et al., 2020). Under this circumstance, early and adequate anti-infective treatment is crucial in severe pneumonia management (Garnacho-Montero et al., 2018). However, traditional pathogen detection methods (culture or specific tests for certain pathogens) seem inadequate for timely comprehensive pathogen identification.

Metagenomic next-generation sequencing (mNGS), an unbiased hypothesis-free detection method, extracts all nucleic acids (DNA or RNA) directly from samples and compares them to reliable database (Chiu and Miller, 2019). Given the broader coverage it provides and the shorter time it requires (Li et al., 2021), mNGS may be valuable in assisting in the diagnosis of rapidly progressing severe pneumonia.

This report describes a case of sCAP caused by *Influenza A virus* and methicillin-resistant *Staphylococcus aureus* (MRSA), which later progressed to hospital-acquired pneumonia and blood stream infection (BSI), suggesting the powerful support of mNGS in rapidly progressing severe pneumonia.

## Case presentation

**Figure 1:**
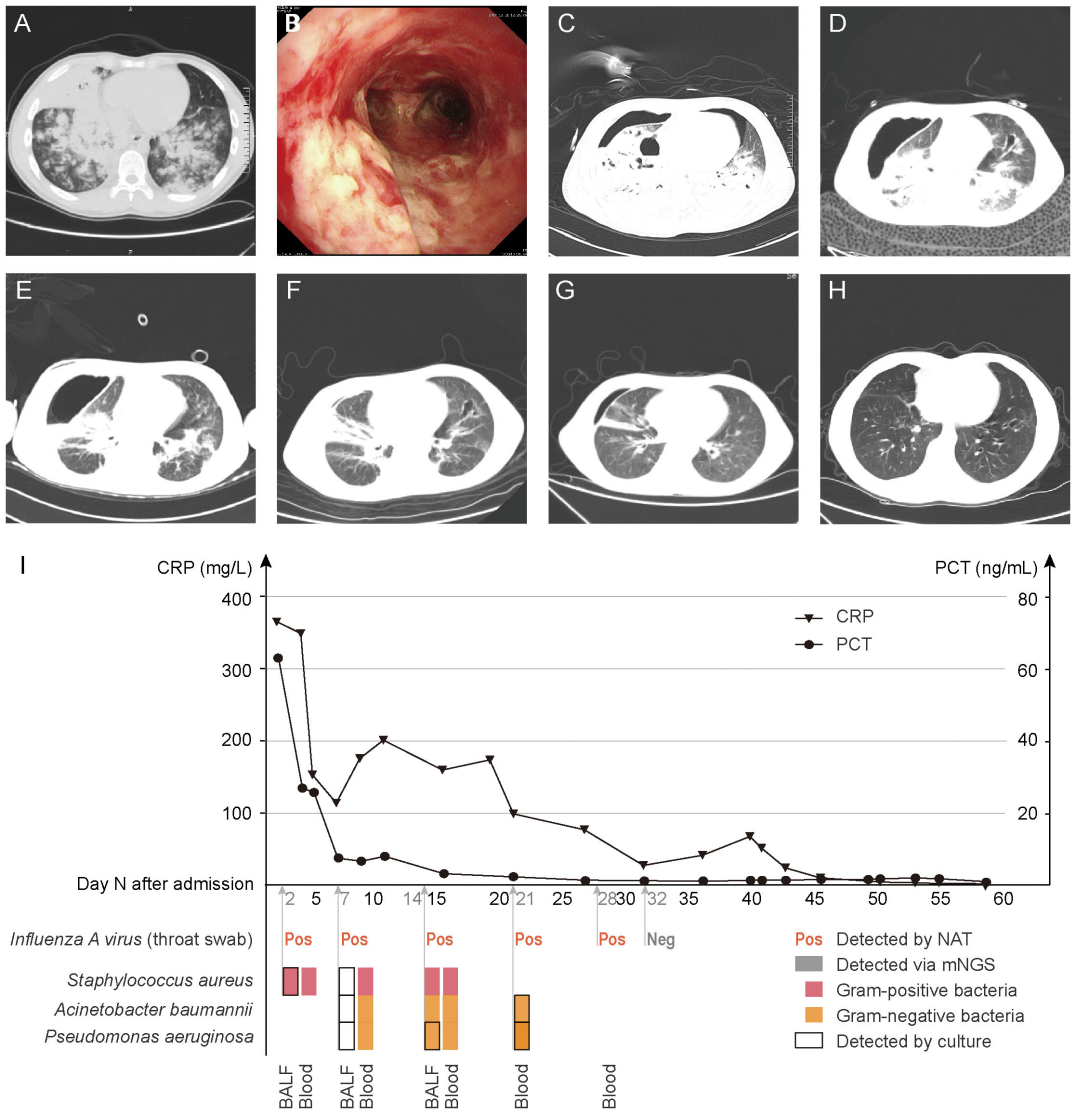
Chest CT and bronchoscopy manifestations, inflammatory indicators and pathogen detection results. **(A)**, 3 days before admission, chest CT. **(B)**, Day 2, bronchoscopy. **(C–G)**, Day 5, 14, 21, 36, 57, chest CT. **(H)**, 6 months after discharge, chest CT. **(I)**, Variation of inflammatory indicators (CRP and PCT) (above horizontal axis). Results of pathogen detections (bellow horizontal axis). BALF and blood were collected on Day 2, 7 and 14; only blood was collected on Day 21 and 32, which were all sent for DNA mNGS and culture. Throat swab was sent for PCR testing for *Influenza A virus*. CRP, C-reactive protein, normal range, <5.00 mg/L. PCT, procalcitonin, normal range, <0.046 ng/mL. BALF, bronchoalveolar lavage fluid.

A 15-year-old Chinese male developed fever (up to 40.0°C), cough and expectoration 7 days prior to admission (December 23, 2019), while symptomatic treatment at the school clinic was not effective. Three days before admission (December 27, 2019), symptoms worsened, chest tightness and dyspnea occurred. Chest CT showed consolidation in the middle lobe of the right lung and nodular shadows in the lower lobes of both lungs (**Figure 1A**). Therefore, he was diagnosed with “sCAP” at the local hospital and was treated with piperacillin. However, his dyspnea aggravated heavily and blood pressure dropped suddenly. After receiving vasopressors and high-flow nasal oxygen (HFNO), he was transferred to our hospital for sepsis shock on December 29, 2019.

On admission, he had an increased respiratory rate (24 breaths/min), increased heart rate (127 beats/min), unstable blood pressure (122/69 mmHg, vasopressor applying), and decreased oxygen saturation (92%, HFNO, 6 L/min). Laboratory investigations showed: white blood cell count, 1.7×10^9^ cells/L; hemoglobin content, 122.5 g/L; platelet count, 77×10^9^ cells/L; partial pressure of carbon dioxide, 29.0 mmHg; partial pressure of oxygen, 56.0 mmHg (HFNO, 6 L/min); blood lactate, 3.0 mmol/L; procalcitonin, 61.69 ng/mL; C-reactive protein, 363.33 mg/L; erythrocyte sedimentation rate, 77.0 mm/h; *Influenza A virus* based on PCR testing of throat swab, positive. On Day 2 of hospitalization, fiberoptic bronchoscopy was performed. A large amount of pseudomembranous necrosis was seen in the airway (**Figure 1B**), and then paired bronchoalveolar lavage fluid (BALF) and peripheral blood were sent for culture and mNGS. Taking the progression rate, epidemiologic characteristics and bronchoscopic manifestations into consideration, he was clinically diagnosed with sCAP caused by influenza virus and Aspergillus. Therefore, we started empirical intravenous anti-infective therapy (peramivir 0.15 g every 24 h, voriconazole 0.20 g every 12 h and biapenem 0.30 g every 6 h) and inhalation of amphotericin B 25 mg every 12 h.

On Day 3, symptoms deteriorated dramatically with an increased respiratory rate (40 breaths/min), increased heart rate (150 beats/min), increased blood pressure (170/90 mmHg) and he developed confusion. To maintain oxygenation, non-invasive ventilation (NIV) through facemasks, nasotracheal intubation were applied successively, however, the patient remained hypoxic. Therefore, he was transferred to ICU for veno-venous extracorporeal membrane oxygenation (VV-ECMO) support. After the immediate and successful application of ECMO, the vital signs stabilized and consciousness was regained. Simultaneously, mNGS results were obtained within 24 h: *Staphylococcus aureus* was detected in both BALF and peripheral blood. Regarding to the mNGS results, intravenous vancomycin 0.40 g every 6 h was given, while antifungal drugs were discontinued gradually. As a result, the patient’s inflammatory markers showed a decline. Subsequently, the diagnosis of MRSA was affirmed by BALF culture after an additional 36 h. On Day 5, chest CT indicated right pneumothorax (**Figure 1C**), so closed thoracic drainage was performed, which successfully drained yellow pus.

On Day 7, in order to assess the effectiveness of the treatment, a second pair of BALF and peripheral blood was sent for mNGS and culture respectively, only a small number of sequences were reported via BALF mNGS, but some Gram-negative bacteria were newly detected via blood mNGS. It rose doubts whether the blood sample was contaminated, so anti-infective drugs were not adjusted immediately. However, *Acinetobacter baumannii* and *Pseudomonas aeruginosa* were newly cultured in BALF culture, and the CRP and PCT levels increased (**Figure 1I**). On Day 14, the body temperature had risen to 38.3°C. Although the abundance of *Staphylococcus aureus* in BALF mNGS and blood mNGS was low, it was cultured in the pleural fluid, linezolid 0.60 g every 12 h was applied. On Day 21, *Acinetobacter baumannii* and *Pseudomonas aeruginosa* were detected via blood mNGS, which were later confirmed by blood culture. Therefore, we gradually adjusted the antibiotic regimen to piperacillin-tazobactam 4.5 g every 8 h, aztreonam 1.0 g every 8 h and polymyxin 50 WIU every 24 h. The inflammatory markers decreased (**Figure 1I**), the pulmonary inflammation alleviated (**Figures 1C–G**) and the symptoms improved.

On Day 19 and Day 32, ECMO and intubation were weaned respectively. On Day 64, the patient was able to engage in simple physical activities and was soon discharged to study in school soon later. After 6 months, a repeat chest CT showed that the exudation was absorbed completely and the inflammation resolved well, with only a small amount of bronchiectasis remaining (**Figure 1H**).

## Discussion

sCAP is the most life-threatening form of CAP, characterized as ICU admission and high mortality (Cavallazzi et al., 2020). With advances in viral detection methods, the role of the virus in CAP has been increasingly recognized (Xu et al., 2020). Influenza is caused by *influenza A* and *influenza B* viruses, characterized as seasonal and population-based, with most of the severe infections occurring in very young or elderly patients (Krammer et al., 2018). Co-infection with bacteria is one of the main reasons for the high pathogenicity and mortality of Influenza. MRSA is one of the pathogens outside the core microorganisms of CAP, its incidence in CAP is low (Torres et al., 2019). However, in recent years, community-associated MRSA (CA-MRSA) infections in healthy young individuals have emerged, and linezolid is recommended for CA-MRSA cases (He and Wunderink, 2020). Unfortunately, these patients being admitted to ICU are vulnerable to hospital-acquired pneumonia (HAP) and other infections, due to severity of illness and exposure to multidrug-resistant (MDR) organisms, which leads to a worse prognosis (Zaragoza et al., 2020).

Prompt initiation and adequate dose of anti-infective treatment is of great importance in severe pneumonia (Garnacho-Montero et al., 2018; Markwart et al., 2020). In the beginning of the treatment, empirical therapy without definite pathogen detection results is feasible and recommended (Zaragoza et al., 2020). However, there are possible omissions in empirical treatment, the support of pathogen detections is necessary. However, culture takes a long time and is unable to detect viruses, PCR testing of specific pathogens relays on the suspicion of clinicians, relatively rare pathogens are likely to be missed, so they are limited in such critical situation. Compared to mNGS and PCR testing, mNGS shows advantages in rapid pathogen detection for serious infectious diseases. With continuous improvement, the sensitivity and specificity of mNGS have improved and the time required has decreased (Cheng et al., 2022). In this case, the patient is a 15-year-old male with no known diseases, relevant family history or bad habits. The co-infection of *Influenza A virus* and MRSA in such a healthy male is relatively rare in clinical management, so we initially ignored the possibility of MRSA but we adjusted the anti-infective therapy timely according to mNGS results within 24 h. Subsequently, mNGS rapidly indicated HAP and bloodstream infection (BSI), the short turnaround time and broad organism spectrum of mNGS greatly saved the time and resulted in satisfactory outcome. Along with the high sensitivity comes unsatisfactory specificity, the possibility of colonization and contamination should be alarmed and it is important to note the potential risk of overuse of broad-spectrum antibiotics. Simultaneously, false-negative results due to unqualified samples or faulty algorithms must also be considered. Therefore, anti-infective drugs should be adjusted according to a comprehensive consideration of physical condition and other indicators.

Additionally, prompt ECMO application for patients with severe pneumonia and acute respiratory distress syndrome (ARDS) can improve the survival rate (Park et al., 2019). And research from other institutions suggested that patients with younger age (less than 45 years) and influenza-related ARDS benefit more from ECMO (Dancer, 2014). The patient was a 15-year-old high school student with influenza. On Day 2 of admission, his condition deteriorated rapidly, so he was immediately transferred to ICU for ECMO support. The application of ECMO allowed time for anti-infective treatment, which is also the main reason for the patient’s recovery.

The mortality of severe influenza pneumonia complicated by MRSA infection is very high. Prompt pathogen detections, timely medication adjustments, powerful ECMO application and active family compliance contribute to this miracle of life.

## Data availability statement

The datasets presented in this study can be found in online repositories. The names of the repository/repositories and accession number(s) can be found below: https://www.ncbi.nlm.nih.gov/, PRJEB61932.

## Ethics statement

The studies involving humans were approved by the Research Ethics Board of First Affiliated Hospital of Zhengzhou University. The studies were conducted in accordance with the local legislation and institutional requirements. Written informed consent for participation in this study was provided by the participants’ legal guardians/next of kin. Written informed consent was obtained from the minor(s)’ legal guardian/next of kin for the publication of any potentially identifiable images or data included in this article. Written informed consent was obtained from the participant/patient(s) for the publication of this case report.

## Author contributions

YL analyzed the data and draft the manuscript. JL collected the clinical data. XM participated in medical care and contributed to the interpretation of the patient data. ZR advised on the research ideas for this study. All authors contributed to the article and approved the submitted version.
