# Supplementary material for: Case Report: Metagenomic next-generation sequencing assists in dynamic pathogen monitoring: powerful tool for progressing severe pneumonia
Source: Front Cell Infect Microbiol. 2023 Sep 4;13:1230813. doi: 10.3389/fcimb.2023.1230813 (PMC10512180; doi:10.3389/fcimb.2023.1230813)
Supplement: Supplementary file 1 [file DataSheet_1.docx]

Supplementary Material

Case Report: Metagenomic Next-generation Sequencing Assists in Dynamic Pathogen Monitoring: powerful tool for progressing severe pneumonia

Yaoguang Li, Jun Lei, Zhigang Ren*, Xiaoxu Ma*

*** Correspondence:** Xiaoxu Ma, [fccmaxx@zzu.edu.cn](mailto:fccmaxx@zzu.edu.cn); Zhigang Ren, [fccrenzg@zzu.edu.cn](mailto:fccrenzg@zzu.edu.cn)

**1. Methods**

Peripheral blood samples (5-6ml per patient) were collected by trained nurses and centrifuged at 4˚C at 1500 rpm for 10 minutes for plasma separation. Then they were centrifuged at 4˚C at 12000 rpm for 10 minutes to eliminate any remaining cells after being carefully collected and transferred the plasma layer to a fresh tube. Following the manufacturer's instructions, cell-free DNA (cfDNA) was extracted from plasma samples using the TIANamp Micro DNA DP316 Kit (Tiangen Biotech, Beijing, China), and RNA was extracted using the TIANamp Virus RNA DP315-R Kit (Tiangen Biotech, Beijing, China).

DNA libraries were constructed using the extracted cfDNA samples and the VAHTS Universal DNA Library Prep Kit V3 for Illumina® (Vazyme, Nanjing, China). RNA libraries were prepared from the extracted RNA samples using the VAHTS Universal RNA-seq Library Prep Kit V6 for Illumina® (Vazyme, Nanjing, China). Each library was set up following its owner's instructions. The Agilent 2100 Bioanalyzer (Agilent Technologies, Santa Clara, USA) was used for library quality control. All libraries were pooled with other libraries using different index sequences and sequenced on an Illumina NextSeq 550Dx platform with the single-end 75bp sequencing option. To monitor the reagent and lab background for each run, no template control (NTC) samples (Nuclease-free H_2_O) were additionally pooled.

Fastq-format data were obtained for each sample by bcl2fastq software (v2.20.0.422, parameters used: --barcode-mismatches 0 --minimum-trimmed-read-length 50). Adapt sequences and low-quality reads were filtered out using cutadapt v2.10 (-q 25, 25 -m 50). The remaining high-quality reads were first mapped to the human genome (hg38, https://hgdownload.soe.ucsc.edu/downloads.html#human) using bwa-mem 2 v2.1 with default parameters, all unmapped reads were then aligned to the NCBI nt database (https://ftp.ncbi.nlm.nih.gov/genomes/) by BLAST v2.9.0+ (-task megablast -num_alignments 10 -max_hsps 1 -evalue 1e-10). Alignments have to be full-length and have a minimum identity of 95%. A customized Python script was used to identify species-specific alignments. Only the alignments that fulfill the above-mentioned criteria were used for further pathogen identification. Pathogens were reported if the following criteria were met: (1) the microbe had at least 3 non-redundant, mapped reads per 10 million raw sequence reads; (2) the microbe was known to be potentially pathogenic in the given clinical context of each patient. The turnaround time is 13 h.

Microbial reads identified from a library were reported if met following criteria: 1) the sequencing data passed quality control filters (library concentration > 50 pM, Q20 > 85%, Q30 > 80%); 2) the species were different from the negative control (NC) of the same sequencing run or the ratio of RPM (sample) to RPM (NC) reached the cutoff that can discriminate true-positives from contaminations and backgrounds (RPM (sample) / RPM (NC)≥5) .

**2. Supplementary Tables**

Supplementary Table 1: Sample Sequencing Characteristic

| Sampling day | Sample type | Raw sequences | Host sequences | lowQ sequences | Clean sequences |
| --- | --- | --- | --- | --- | --- |
| Day 2 | BALF | 16992356 | 16192570 | 8268 | 791518 |
| Day 2 | Blood | 43186741 | 42055585 | 27922 | 1103234 |
| Day 7 | BALF | 17755857 | 17393298 | 3518 | 359041 |
| Day 7 | Blood | 28493577 | 27460449 | 24558 | 1008570 |
| Day 14 | BALF | 17876901 | 17247102 | 4492 | 625307 |
| Day 14 | Blood | 18378310 | 17992083 | 11875 | 374352 |
| Day 21 | Blood | 87869043 | 85620216 | 125930 | 2122897 |
| Day 60 | Blood | 21655812 | 21226892 | 5256 | 423664 |
| Day 187 | Blood | 22111006 | 20481239 | 544 | 1629223 |

BALF, bronchoalveolar lavage fluid.

Supplementary Table 2: mNGS Results for Pathogen Detection

| Sampling day | Sample type | Organisms detected via mNGS | Sequences |
| --- | --- | --- | --- |
| Day 2 | BALF | *Staphylococcus aureus* | 24221 |
|  |  | *Haemophilus influenzae* | 71 |
|  |  | *Streptococcus pneumoniae* | 48 |
| Day 2 | Blood | *Staphylococcus aureus* | 3431 |
|  |  | *Haemophilus influenzae* | 193 |
|  |  | *Streptococcus pneumoniae* | 213 |
| Day 7 | BALF | *Acinetobacter Joni* | 56 |
|  |  | *klebsiella pneumoniae* | 56 |
|  |  | *Streptococcus pneumoniae* | 26 |
| Day 7 | Blood | *Staphylococcus aureus* | 8055 |
|  |  | *Pseudomonas aeruginosa* | 4657 |
|  |  | *Acinetobacter baumannii* | 1845 |
|  |  | *Acinetobacter Joni* | 442 |
|  |  | *Hemophilus parainfluenzae* | 69 |
| Day 14 | BALF | *Pseudomonas aeruginosa* | 77664 |
|  |  | *Acinetobacter baumannii* | 3890 |
|  |  | *Hemophilus parainfluenzae* | 634 |
|  |  | *Stenotrophomonas maltophilia* | 300 |
|  |  | *Burkholderia cepacia* | 219 |
|  |  | *Staphylococcus aureus* | 51 |
|  |  | *Streptococcus pneumoniae* | 6 |
| Day 14 | Blood | *Pseudomonas aeruginosa* | 4865 |
|  |  | *Acinetobacter baumannii* | 678 |
|  |  | *Escherichia coli* | 83 |
|  |  | *Hemophilus parainfluenzae* | 22 |
|  |  | *Staphylococcus aureus* | 152 |
|  |  | *Human herpesvirus 6* | 2 |
| Day 21 | Blood | *Aeromonas caviae* | 6641 |
|  |  | *Pseudomonas aeruginosa* | 2068 |
|  |  | *Acinetobacter haemolyticus* | 813 |
|  |  | *Acinetobacter baumannii* | 201 |
|  |  | *Klebsiella pneumoniae* | 234 |
|  |  | *Hemophilus parainfluenzae* | 28 |
|  |  | *Streptococcus parasanguis* | 46 |
|  |  | *Streptococcus mitis* | 21 |
|  |  | *Human herpesvirus 6* | 1 |
| Day 60 | Blood | *Torque teno virus* | 230 |
| Day 187 | Blood | *Not detected* | - |

BALF, bronchoalveolar lavage fluid.
